# Cross-variant proof predictive vaccine design based on SARS-CoV-2 spike protein using immunoinformatics approach

**DOI:** 10.1186/s43088-023-00341-4

**Published:** 2023-01-10

**Authors:** Lavanya Kumar Sahu, Kiran Singh

**Affiliations:** Department of Biological Sciences and Engineering, Maulana Azad National Institute of Technology, Bhopal, Madhya Pradesh India

**Keywords:** Multi-epitope vaccine, Immunoinformatics, SARS-CoV-2, COVID-19, Delta, And omicron

## Abstract

**Background:**

Coronavirus Disease (COVID-19) is caused by the Severe Acute Respiratory Syndrome Coronavirus 2 (SARS-CoV-2). The SARS-CoV-2 virus is evolving continuously. The omicron variant of SARS-CoV-2 has the highest mutation in its spike protein, thus making the presently available vaccine ineffective or reducing its efficiency. Furthermore, the majority of the vaccines are constructed using a spike protein sequence from wild-type SARS-CoV-2. This raises the possibility of the virus evolving to the point where the vaccine's effectiveness is completely lost, even after booster doses. The study aims to develop a predictive vaccine as well as the epitopes for the updating of the vaccine sequences of currently available vaccines. In this study, following the immunoinformatics approach, predictive vaccine construction was done with the help of epitopes present on spike proteins of wild-type, delta, and omicron variants that encompass the majority of variants and possible new variants that arise from the combination of circulating variants.

**Results:**

The vaccine that was constructed was stable and immunogenic. The vaccine was constructed with the help of 18 B-cell epitopes, 5 MHC class I epitopes, and 6 MHC class II epitopes. The epitope conservancy analysis suggests that the vaccine will work for the previously known variant of concern. The vaccine bound to TLR4, TLR2, B-cell receptor chains A and B, and ACE2 receptors with a *z* score of − 1.4, − 1.7, − 1.4, − 1.7, and − 1.4, respectively, with a cluster size of 121 highest for the ACE2 receptor and 46 lowest for B-cell receptor chain A. The C-ImmSim simulation results indicate that the vaccine is generating both humoral and cell-mediated responses at a sufficient level throughout the month upon injection of the vaccine as an antigen.

**Conclusion:**

The study's findings indicate that the vaccine was both stable and immunogenic, providing a sufficient level of immunity. Following experimental validation, the vaccine can be used, and the epitopes can be employed for therapeutic purposes such as antibody synthesis.

**Graphical Abstract:**

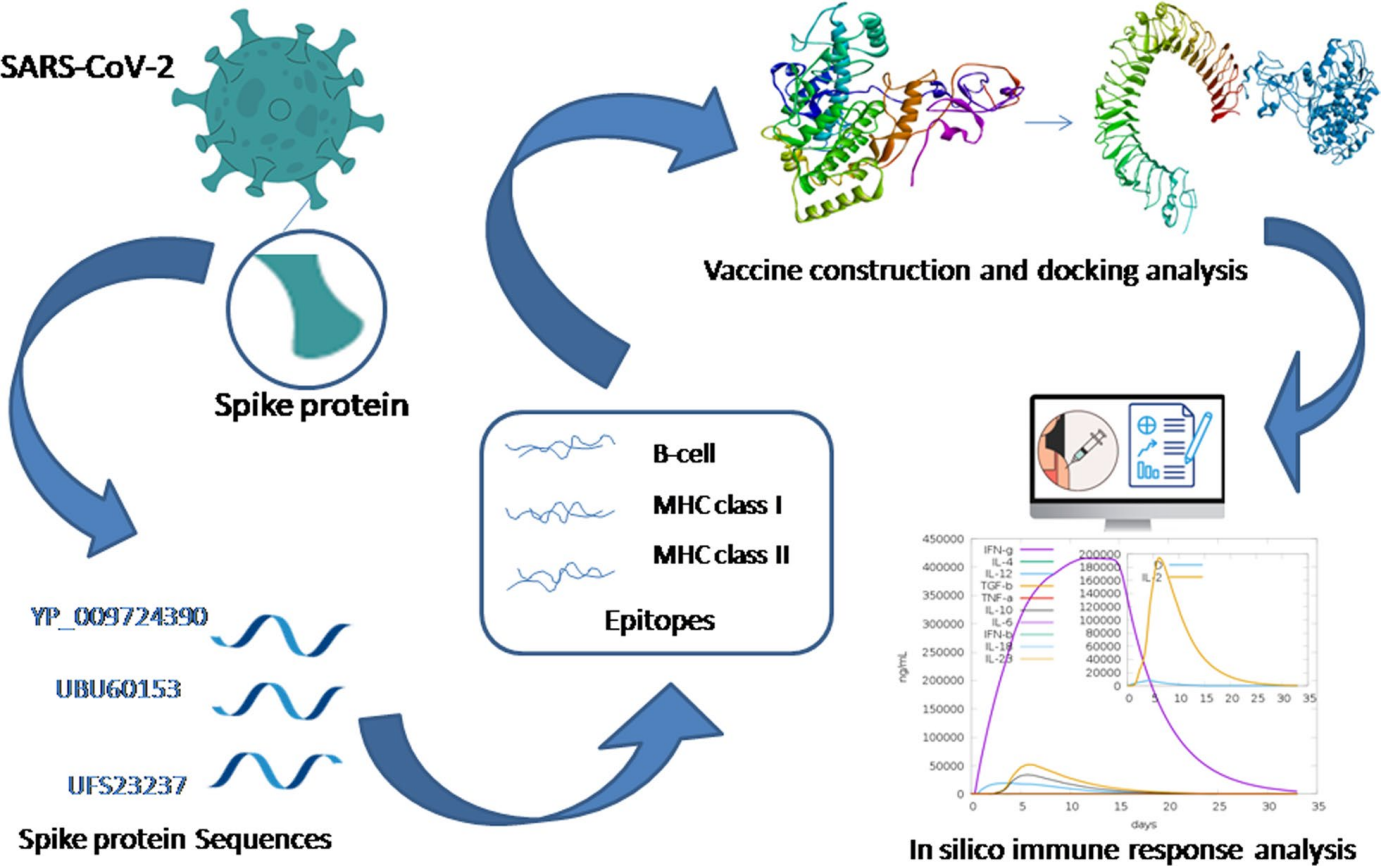

**Supplementary Information:**

The online version contains supplementary material available at 10.1186/s43088-023-00341-4.

## Background

A novel coronavirus was identified in Wuhan, China, in December 2019; it is termed Severe Acute Respiratory Syndrome Coronavirus-2 (SARS-CoV-2) [1], which is the cause of the current pandemic. The disease was named Coronavirus Disease (COVID-19) by the World Health Organisation (WHO). The SARS-CoV-2 has a positive-sense RNA genome. Although SARS-CoV-2 has proof-reading capability due to which mutations are comparatively lesser than other RNA viruses, it also has a small proportion of mutations that affect the functional properties and alter the infectivity, disease severity, or interaction with the host immune system. These were proven with the classification of “Variant of Concern (VOC)” for the newly emerging strains of SARS-CoV-2 in late 2020.

The previously known variant of concern alpha (B.1.1.7) with mutation N501Y in the Receptor Binding Domain (RBD) of spike protein improved the affinity towards human the Angiotensin Converting Enzyme 2 (ACE2) receptor. Alpha also has the P681H mutation that affects the infectivity and replication of the virus, as well as mutations in the N-Terminal Domain (NTD) D178H, HV69-70del, and Y144del that affect the neutralisation by NTD-directed antibodies as they result in a conformational change in the NTD of spike protein [2]. Beta (B.1.351) and Gamma (P.1) have the mutations N501, E484K, and K417T/N that affect the binding affinity towards ACE2 receptor, and K417T/N alone was believed to decrease the affinity while E484K and N501Y were increasing the affinity whereas, N501Y alone increase the affinity by 10-folds [3]. E484K is also capable of significant antibody evasion. While other variants have N501Y, E484K mutations that increase SARS-CoV-2 spike protein affinity towards the ACE2 receptor, delta (B.1.617.2) has a completely different set of RBD mutations: D614G, L452R, and T478K that have a higher affinity towards the ACE2 receptor. Moreover, L452R and T478K showed more affinity towards the ACE2 receptor compared with other mutations [4] along with P681R, which helps cleave the precursor spike protein, thus promoting superior binding compared to others [5]. Delta variant L452R was also proved to evade antibody neutralisation as well as cellular immunity [6, 7]. Epsilon (B.1.427/B.1.429) has several mutations; however, only 3 mutations are considered signature mutations: S13I, W152C, and L452R. These are involved in antibody evasion, and the S13I is a mutation in the signal peptide, and the W152C is a mutation in NDT. These were responsible for the complete loss of NTD neutralisation for monoclonal antibodies targeting the NTD region [8]. The current variant of concern Omicron and its sub-lineages B.1.1.529, BA.1, BA.1.1, BA.2, BA.3, BA.4, and BA.5 have several mutations, and numerous studies have shown that they evade most of the antibodies and reduce the neutralisation activity of those antibodies that are still effective.

There were vaccine efficiency data and shreds of evidence of reduced neutralisation of some SARS-CoV-2 variants by post-vaccination sera. However, a deep understanding of correlates of protection was required to evaluate how this may impact vaccine effectiveness. Nonetheless, a possible update of the vaccine sequence is required for better immunity from the existing as well as emerging variants [2]. Andrews et al. [9] showed that vaccine effectiveness against symptomatic disease with the Omicron variant is significantly lower than with the Delta variant and a booster dose is required, booster dose of BNT162b2 for ChAdOx1-nCoV-19, BNT162b2, and mRNA-1273 increased the vaccine’s effectiveness to 62.4%, 67.2%, and 64.9%, respectively. As for the having mRNA-1273 as a booster dose for ChAdOx1-nCoV-19, BNT162b2 and mRNA-1273 increased the vaccine’s effectiveness to 70.1%, 73.9%, and 66.3%, respectively. In both cases, the vaccine’s effectiveness was waning after 3 weeks [9]. The efficiency of vaccines was reduced for the new variant (delta), and a booster dose was required to increase the efficiency. This seems to be sufficient to a degree in stating the need for vaccine sequence update.

In this study, a predictive multi-epitope vaccine was designed from the spike protein sequence of wild-type, delta, and omicron variants to cover a wide range of known and/or emerging variants. The multi-epitope vaccine has various epitopes to form the vaccine, giving it the advantage to work on multiple targets. These epitopes are the key factor that can help to update the vaccine sequence by providing data like antigenicity, population coverage, and conservation of these peptides in the spike protein sequences. This study focuses on spike protein and its sequences as they were the primary factor responsible for the interaction with the host (human) as well as for the construction of the vaccines. In addition, the emergence of new variants was facilitated by the mutations of spike protein sequences.

## Methods

### Sequence retrieval

The spike glycoprotein sequences were obtained from NCBI Virus (https://www.ncbi.nlm.nih.gov/labs/virus/vssi/#/sars-cov-2). NCBI Virus is a centralised repository for viral sequence data, which includes RefSeq, GenBank, and other NCBI repositories. There is also a SARS-CoV-2 data hub. It was used to obtain the spike protein sequences of SARS-CoV-2 wild-type, delta, and omicron variants.

### Epitope prediction

For the prediction of the linear B-cell epitopes, the ABCpred server was used (https://webs.iiitd.edu.in/raghava/abcpred/ABC_submission.html), which is an Artificial Neural Network (ANN)-based B-cell epitope prediction server. It predicts B-cell epitope with an accuracy of 65.93% in an antigen sequence based on a partial recurrent neural network in a fixed length pattern of 10, 12, 14, 16, 18, and 20 [10]. The epitopes were predicted for multiple lengths: 10, 12, 14, 16, 18, and 20.

The T-cell epitopesare classified as cytotoxic T lymphocytes epitopes (MHC class I) and helper T lymphocytes epitopes (MHC class II) and are used to induce specific immune responses which are based on CD4^+^ and CD8^+^ T-cells. To predict these HLA binding epitopes, TepiTool (http://tools.iedb.org/tepitool/) of the Immune Epitope DataBase (IEDB) was used. TepiTool is a 6-step wizard that provides several top MHC binding prediction algorithms with hundreds of alleles for different species and, as a wizard, it provides a user-friendly interface with optimal cutoffs and guidelines [11]. For the prediction of T cell epitopes, all 3 sequences were given as input query sequences simultaneously.

A set of 12 MHC class I super-types was chosen for MHC class I binding epitope prediction, and the peptide length was set to 9 mers. The prediction method used was consensus, and the predicted peptide was chosen based on the predicted percentile rank with a cutoff value ≤ 1. Following that, the same procedure was repeated with a change in the predicted peptide method as the IC50 value with a cutoff of ≤ 500 nM. For MHC class II binding epitopes, the peptide length was fixed at 15 mers, the prediction method was IEDB recommended, and the predicted peptide was selected using the 7-allele method [12] which represents a peptide as a good binder based on its median consensus percentile rank with a cutoff value of ≤ 20 with default parameters, the lower the median percentile rank, the better the binder.

### Epitope screening and conservancy analysis

Epitope screening is the process of filtering previously procured epitopes to identify felicitous epitopes. In this elimination of duplicates, the removal of allergen and toxin peptides was done, while the most antigenic epitopes were preferred. This procedure is followed for both B-cell and T-cell epitopes. The Vaxijen server was used for antigenicity propensity analysis of epitopes. Vaxijen 2.0*v* (http://www.ddg-pharmfac.net/vaxijen/VaxiJen/VaxiJen.html) a server to calculate antigenicity, has an accuracy of 70–89% [13]. The threshold was set to 0.5 for an epitope to be antigenic and used to calculate the antigenicity propensity score. The Algpred server was used to identify allergen and non-allergen epitopes. Algpred 2.0 (https://webs.iiitd.edu.in/raghava/algpred2/batch.html) is a server to identify allergen and non-allergen peptides. It was based on a machine learning algorithm to predict allergenic and non-allergenic proteins [10]. The threshold was set at 0.3 for a peptide to be an allergen, and non allergen peptides were selected. To identify toxic and non-toxic peptide epitopes. The ToxinPred server was employed. The ToxinPred (https://webs.iiitd.edu.in/raghava/toxinpred/multi_submit.php) is a server for the prediction and designing of toxic and non-toxic peptides. It has 1805 toxic peptides with less than or equal to 35 residues [14]. The threshold was set to be 0.0 in the Support Vector Machine (SVM) method for a peptide to be a toxin, and non-toxic peptides were selected.

In addition to antigenicity, non-allergenicity, and non-toxicity, the T-cell epitopes require additional properties to be a suitable candidate, i.e. immunogenicity and interferon-gamma induction (INF-γ) to be used for vaccine construction. The IEDB Class I immunogenicity tool was used for immunogenicity analysis to predict MHC I immunogenicity [15]. The immunogenicity of a peptide MHC complex is predicted by the properties and position of amino acids in the sequence. This tool was validated for peptides of 9 mer length, although it can also be used for peptides of any length. The threshold for a peptide to be immunogenic was set to zero, and the CD4 T cell immunogenicity prediction tool of the IEDB was used to predict MHC II immunogenicity. In this tool, the 7-allele method was used for the prediction [16]. While IFNepitope, a web server for the prediction and design of the epitopes that can induce the release of interferon-gamma [17], is used to analyse IFN- induction, which is only performed for MHC II epitopes.

The IEDB's Epitope conservancy analysis tool was used to determine epitope conservancy. IEDB's epitope conservancy analysis (http://tools.iedb.org/conservancy/) computes the extent of epitopes conserved within the protein sequence at an identity level set by the user [18]. This conservation was performed to ensure that an epitope was conserved in various SAR-CoV-2 variants, primarily the previously VOC-labelled strains alpha, beta, gamma, delta, epsilon, and omicron, with a sequence identity threshold ≥ 100%.

### Population coverage

The T-cell peptide epitopes were used to estimate the patient population targeted by a vaccine based on these epitopes. For this purpose, the population coverage tool of the IEDB (http://tools.iedb.org/population/) was used [19]. A set of 11 epitopes is given as input for the analysis of which 5 are MHC I-binding epitopes and 6 are MHC II-binding epitopes. This analysis was done for the world population.

### Vaccine construction

The vaccine was devised through a sequential arrangement with flexible linkers (EAAAK, KK, and GPGPG) and adjuvant (Beta defensin 2, UniProtKB ac: O15263). The linkers were used to better represent the epitopes as well as improve the immunogenicity and stability of a protein. Adjuvants help in improving immunogenicity and stability. Moreover, beta-defensin has a link to the activation of pathogen-specific innate and adaptive immunity, specifically beta-defensin 2 for enhancing type I immune response [20, 21]. Furthermore, epitope cluster analysis was done to arrange the similar sequences in a group or adjacent to each other with the help of the epitope cluster analysis tool of the IEDB [22].

### Assessment of vaccine construct

#### BLAST against the human proteome

The Basic Local Alignment Search Tool (BLAST) assessment of the vaccine construct against the human proteome was done to find any similar or homologous proteins to avoid any disruption in the human system.

#### Physical, chemical, and physiochemical properties

For the assessment of the vaccine construct, the ProtParam tool (https://web.expasy.org/protparam/) of ExPASy was used to find the physical and chemical properties of the vaccine construct. ProtParam allows the computation of physical and chemical properties of protein stored in SwissProt/TrEMBL or user-provided protein sequences. Furthermore, the Scratch protein predictor was used for (http://scratch.proteomics.ics.uci.edu) SOLpro, DLpro, and ANTIGENpro for solubility upon overexpression, Disulphide bonds, and antigenicity.

#### Secondary structure

For secondary structure prediction, the Self-optimized prediction method with alignment (SOPMA) (https://npsa-prabi.ibcp.fr/cgi-bin/npsa_automat.pl?page=/NPSA/npsa_sopma.html) and PSIPRED tools were used with default parameters [23, 24]. Secondary structure prediction was a key step to better understanding the tertiary structure as well as the nature of the protein.

#### Tertiary structure prediction, refinement, and validation

Tertiary structure prediction of the vaccine construct was done by the Iterative Threading ASSEmbly Refinement (I-TASSER) server, and predicted protein structure was validated with the help of the Ramachandran plot [25–28]. The Ramachandran plot server was used to get the Ramachandran plot [29]. The tertiary structure was refined by the ModRefiner server and was further meliorated by the GalaxyRefiner server [30]. The current top-ranked method for 3D structure prediction is AlphaFold. So, the tertiary structure was also predicted using AlphaFold 2, and the results were compared with I-TASSER’s results.

### Docking analysis and immune response analysis

To test vaccine construct binding affinity with immune receptors, docking analysis was performed with the ACE2 receptor (PDB ID: 3SCI), TLR2 (PDB ID: 2Z7X), TLR4 (PDB ID: 3FXI), and BCR (PDB ID: 3KG5). To verify the binding affinity of vaccine constructs with class I and class II HLAs, the MHC class I HLA A*0201 (PDB ID: 4U6Y), HLA B*5101 (PDB ID: 4MJI), and MHC class II HLA DRB1*1402 (PDB ID: 6ATF) were docked. For docking analysis, the High Ambiguity Driven protein–protein DOCKing (HADDOCK 2.4) server (https://wenmr.science.uu.nl/haddock2.4/) was used [31, 32]. The prediction of binding amino acid residues for each of the receptors and the modelled vaccine construct was done using the Metamethod for Protein–Protein Interaction Site Prediction (meta-PPISP) server (https://pipe.rcc.fsu.edu/meta-ppisp.html) [33].

The C-ImtmSim server (https://kraken.iac.rm.cnr.it/C-IMMSIM/) was used to analyse the immune response generated by a vaccine injection by simulating the immune system [34, 35]. The immune response was checked for the vaccine as antigen. Additionally, a comparison was made between the predictive vaccine and the currently available vaccine based upon the C-ImmSim simulation results by utilising the S-2P protein sequence encoded by Pfizer and Moderna’s COVID-19 vaccine. Surface glycoprotein (YP 009724390.1) with a mutation at positions 986 and 987 was encoded by Pfizer and Moderna’s vaccine. The detailed results of the comparison are available in Additional file 1.

## Results

### Sequence retrieval

NCBI Virus accession numbers for the spike protein sequences were YP 009724390, UBU60153, and UFS23237. The accession number YP_009724390 represents the sequence of spike glycoprotein isolated from Wuhan, China in December 2019; and the UBU60153 represents the sequence of spike glycoprotein of variant B.1.617.2 isolated from India in September 2021; and the UFS23237 represents the sequence of spike glycoprotein of variant B.1.1.529 isolated from Missouri, USA. With the mutations of the delta and omicron variants, the similarity with wild-type sequences is 99.37% and 96.87%, respectively. Due to the high similarity, the epitopes were obtained separately for each sequence, and multiple sequence alignment was avoided.

### Epitopes prediction

The initial output of B-cell epitopes included over 200 epitopes that were manually filtered to remove duplicates from different query inputs as well as overlapping epitopes of different lengths with the same core sequence. Only 18 B-cell peptide epitopes were identified as suitable candidates for vaccine construction after epitope screening of the entire B-cell repertoire. Table 1 shows the epitopes and their conversancy results.

**Table 1:** The B-cell epitopes with antigenicity and conservancy analysis

Epitopes	Antigenicity	Conserved (Y) Not-conserved (N)							Percentage of protein sequence match ≥ 100%	Minimum identity (%)	Maximum identity (%)
		W	δ	O	α	Β	γ	ε			
NKSWMESEFR	0.6813	Y	Y	Y	Y	Y	Y	N	85.71% (6/7)	90.00	100.00
LREFVFKNID	0.8332	Y	Y	Y	Y	Y	N	Y	85.71% (6/7)	90.00	100.00
PDKVFRSSVLHS	1.649	Y	Y	Y	Y	Y	Y	Y	100.00% (7/7)	100.00	100.00
GINITRFQTLLALHRSYLTP	0.6602	Y	Y	Y	Y	N	Y	Y	85.71% (6/7)	50.00	100.00
KTQSLLIVNN	0.8332	Y	Y	Y	Y	Y	Y	Y	100.00% (7/7)	100.00	100.00
MDLEGKQGNFKN	1.3296	Y	Y	Y	Y	Y	Y	Y	100.00% (7/7)	100.00	100.00
KNIDGYFKIYSKHTPINL	0.749	Y	Y	N	Y	Y	Y	Y	85.71% (6/7)	88.89	100.00
FLGVYYHKNNKSWMESEFRV	0.5741	Y	N	N	N	Y	Y	N	42.86% (3/7)	70.00	100.00
LGDIAARDLI	0.9922	Y	Y	Y	Y	Y	Y	Y	100.00% (7/7)	100.00	100.00
HADQLTPTWR	0.6323	Y	Y	Y	Y	Y	Y	Y	100.00% (7/7)	100.00	100.00
TSALLAGTIT	0.6493	Y	Y	Y	Y	Y	Y	Y	100.00% (7/7)	100.00	100.00
IAYTMSLGAE	1.0165	Y	Y	N	Y	N	Y	Y	71.43% (5/7)	90.00	100.00
SIAIPTNFTISVTT	0.8408	Y	Y	Y	N	Y	Y	Y	85.71% (6/7)	92.86	100.00
EIRASANLAATKMS	0.8607	Y	Y	Y	Y	Y	N	Y	85.71% (6/7)	92.86	100.00
KRVDFCGKGYHLMS	1.0454	Y	Y	Y	Y	Y	Y	Y	100.00% (7/7)	100.00	100.00
MSLGAENSVAYSNN	0.8278	Y	Y	N	Y	N	Y	Y	71.43% (5/7)	92.86	100.00
IPFAMQMAYRFNGIGVTQ	1.4137	Y	Y	Y	Y	Y	Y	Y	100.00% (7/7)	100.00	100.00
PTNFTISVTTEILPVSMTKT	1.2666	Y	Y	Y	N	Y	Y	Y	85.71% (6/7)	95.00	100.00

W, δ, O, α, β, γ, and ε correspond to spike protein of wild-type (B), delta (B.1.617.2), omicron (BA.1.17.2), alpha (B.1.17), beta (B.1.351), gamma (P.1) and epsilon (B.1.427) variant of SARS-CoV-2

Based upon the thresholds mentioned for T-cell epitopes, many epitopes are obtained, and after the screening process, 5 MHC class I epitopes and 6 MHC class II epitopes were identified as suitable candidates for vaccine construction. Five MHC class I peptide epitopes were identified, with peptide “AEIRASANL” having the lowest immunogenicity score and IC50 value and peptide “MSLGVENSV” with the highest immunogenicity score and IC50 value, having 0.00689, 9.62, and 0.8819, 466.42 scores, respectively, while six MHC class II peptides were identified, of which “IWLGFIAGLIAIVMV” and “WYIWLGFIAGLIAIV” were found positive for IFN-γ induction, having Median consensus percentile (immunogenicity) at 18 (epitopes separately listed in Additional file 1: Table 2 and 3). The conservancy results indicate all epitopes are conserved in at least 5 variants out of 7 variants except the “LGVYYHKNNKSWMESEFRV” epitope, which is only conserved in wild-type, beta, and gamma variants, and “MSLGVENSV,” which is only conserved in omicron and beta variants while the valine at the 5^th^ position of this peptide sequence was observed to have been replaced by alanine that is presented in all other variants. Table 2 lists epitopes with conservancy results. Furthermore, IEDB was searched for a similar sequence of peptide epitopes identified in this study, and it was found that a peptide epitope “MSLGVENSV” was not reported yet and only a substring was reported from an unidentified source of the epitope.

**Table 2:** The T-cell epitopes (MHC class I and MHC class II; 9mer and 15mer) with antigenicity and conservancy analysis

Epitopes	Antigenicity	Conserved (Y) Not-conserved (N)							Percentage of protein sequence match ≥ 100%	Minimum identity (%)	Maximum identity (%)
		W	δ	O	α	Β	γ	ε			
KWPWYIWLG	1.0478	Y	Y	Y	Y	Y	Y	Y	100.00% (7/7)	100.00	100.00
NRALTGIAV	0.5302	Y	Y	N	Y	Y	Y	Y	85.71% (6/7)	88.89	100.00
IAIVMVTIM	1.1339	Y	Y	Y	Y	Y	Y	Y	100.00% (7/7)	100.00	100.00
AEIRASANL	0.7082	Y	Y	Y	Y	Y	Y	Y	100.00% (7/7)	100.00	100.00
MSLGVENSV	0.6283	N	N	Y	N	Y	N	N	28.57% (2/7)	88.89	100.00
IPFAMQMAYRFNGIG	1.2828	Y	Y	Y	Y	Y	Y	Y	100.00% (7/7)	100.00	100.00
SNLKPFERDISTEIY	0.8255	Y	Y	Y	Y	Y	Y	Y	100.00% (7/7)	100.00	100.00
AEIRASANLAATKMS	0.8255	Y	Y	Y	Y	Y	N	Y	85.71% (6/7)	93.33	100.00
IGINITRFQTLLALH	0.8391	Y	Y	Y	Y	N	Y	Y	85.71% (6/7)	73.33	100.00
WYIWLGFIAGLIAIV	0.577	Y	Y	Y	Y	Y	Y	Y	100.00% (7/7)	100.00	100.00
IWLGFIAGLIAIVMV	0.615	Y	Y	Y	Y	Y	Y	Y	100.00% (7/7)	100.00	100.00

W, δ, O, α, β, γ, and ε correspond to spike protein of wild-type (B), delta (B.1.617.2), omicron (BA.1.17.2), alpha (B.1.17), beta (B.1.351), gamma (P.1) and epsilon (B.1.427) variant of SARS-CoV-2

### Population coverage

The class I and class II MHC epitopes were used for the estimation of the patient population that can be targeted by this predictive vaccine. These peptide epitopes combined cover 94.1% of the world population, where MHC class I peptide epitopes cover 88.42% and MHC class II peptides cover only 49.02%.

### Vaccine construction

**Fig. 1:**
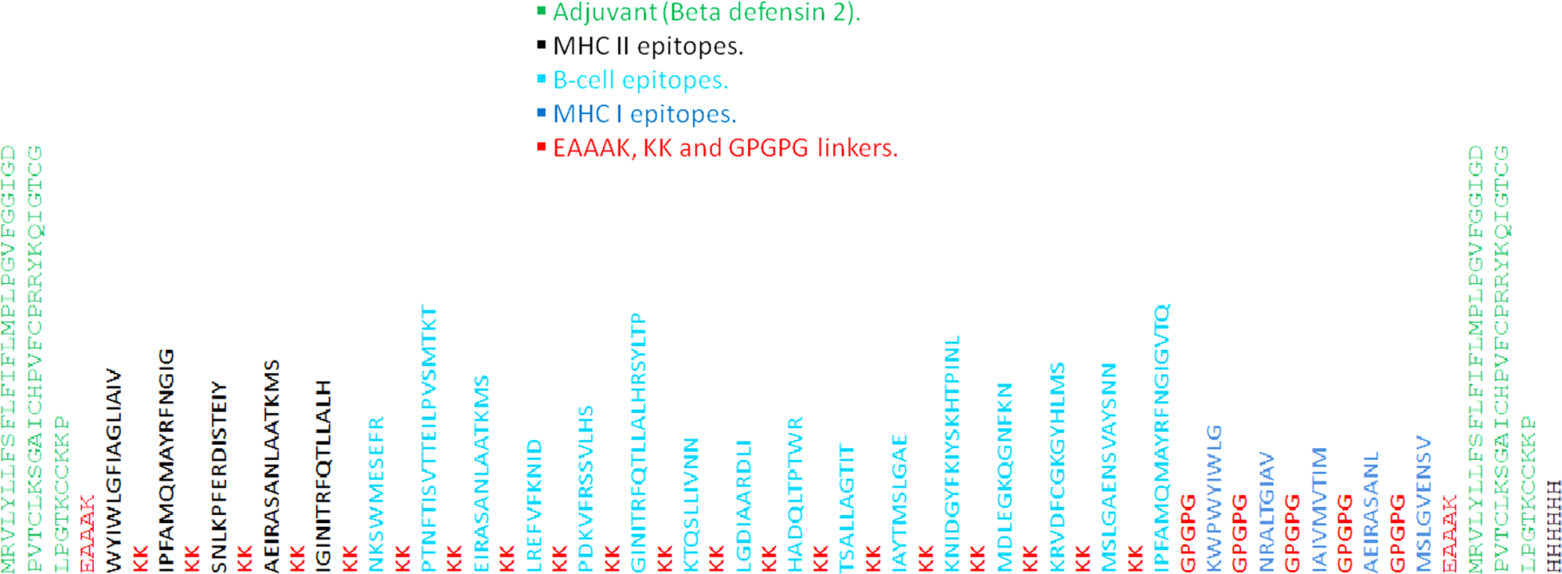
The sequential arrangements of epitopes to form vaccine construct

The vaccine was constructed by sequentially arranging the epitopes, clustered epitopes were arranged adjacent to each other, and singleton epitopes were arranged within the respective category of epitopes. The arrangement starts with adjuvant, followed by MHC II epitopes, B-cell epitopes, MHC I epitopes, and adjuvant, and lastly by the 6x-His tag. All these epitopes, and adjuvants were joined by linkers. Figure 1 depicts the vaccine construct (FASTA format in Additional file 1).

### Assessment of vaccine construct

The BLAST results show 100% identity to Beta defensin 2, with coverage of only 17%, while other proteins’ identities were less than 40%. This is due to the use of adjuvant in vaccine construction that is sourced from the human proteome.

The vaccine construct's general properties computed with the Protparam server have a molecular weight of 59,855.32 Da, the theoretical pI is 10.9, and the number of positively and negatively charged amino acids is 90 and 26, respectively. It thus corresponds to the positively charged nature of the protein. The instability index of 30.60 shows it as stable, as the score for stable protein is less than 40. The Grand average of hydropathicity (GRAVY) of − 0.137 shows it as hydrophilic. The vaccine construct's aliphatic index of 83.88 indicates that it is a highly thermostable protein. The results of SOLpro, DLpro, and ANTIGENpro results for solubility upon overexpression, number of disulphide bonds, and antigenicity were 0.850797, 6, and 0.383958, respectively.

**Fig. 2:**
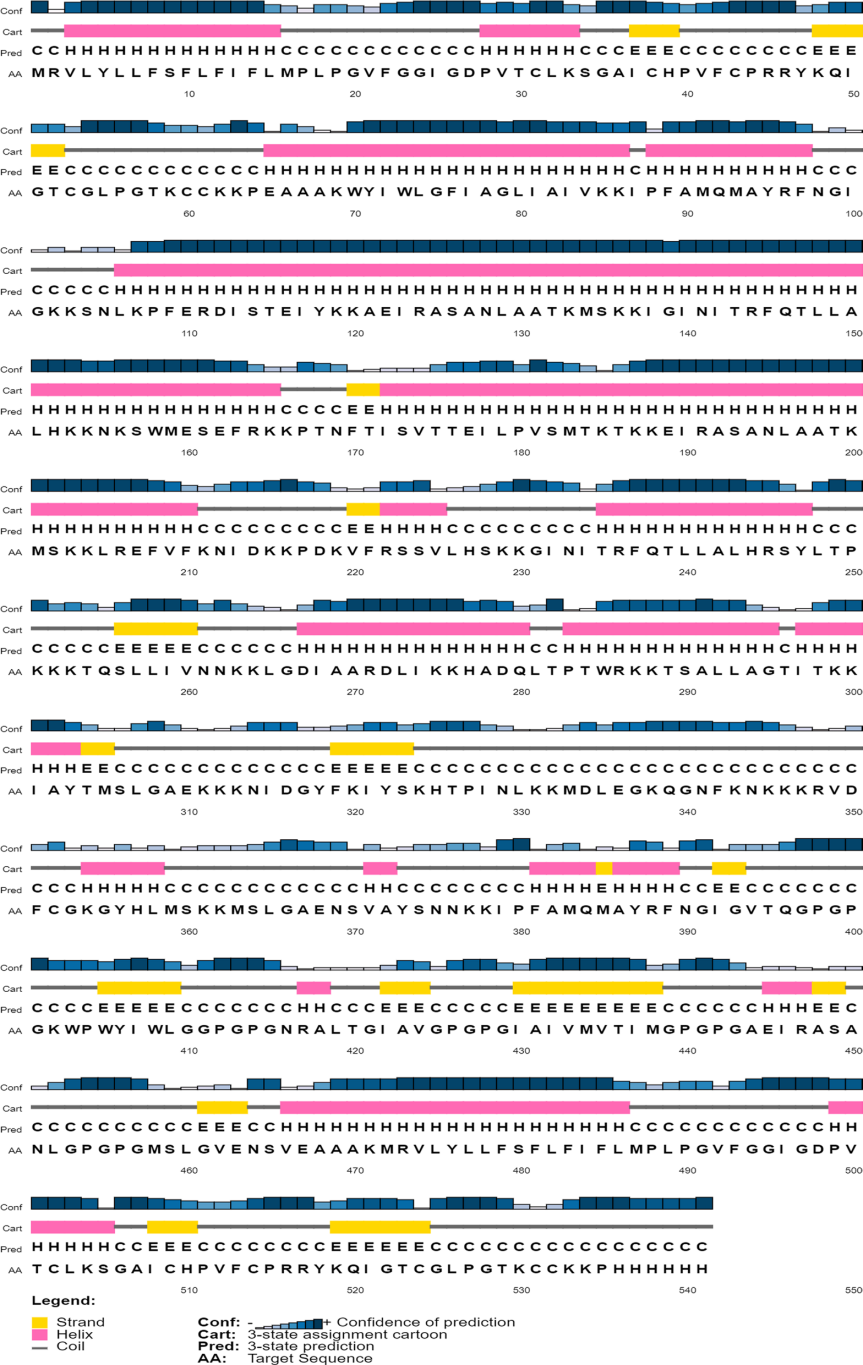
Confidence level of prediction of the secondary structure via PSIPRED

The SOPMA result shows that the construct contains 45.10% alpha helices, 16.45% extended strands, 4.99% beta turns, and 33.46% random coils. Figure 2, obtained using PSIPRED, shows the confidence level of the prediction of secondary structure. Further information on the secondary structure was in Additional file 1.

**Fig. 3:**
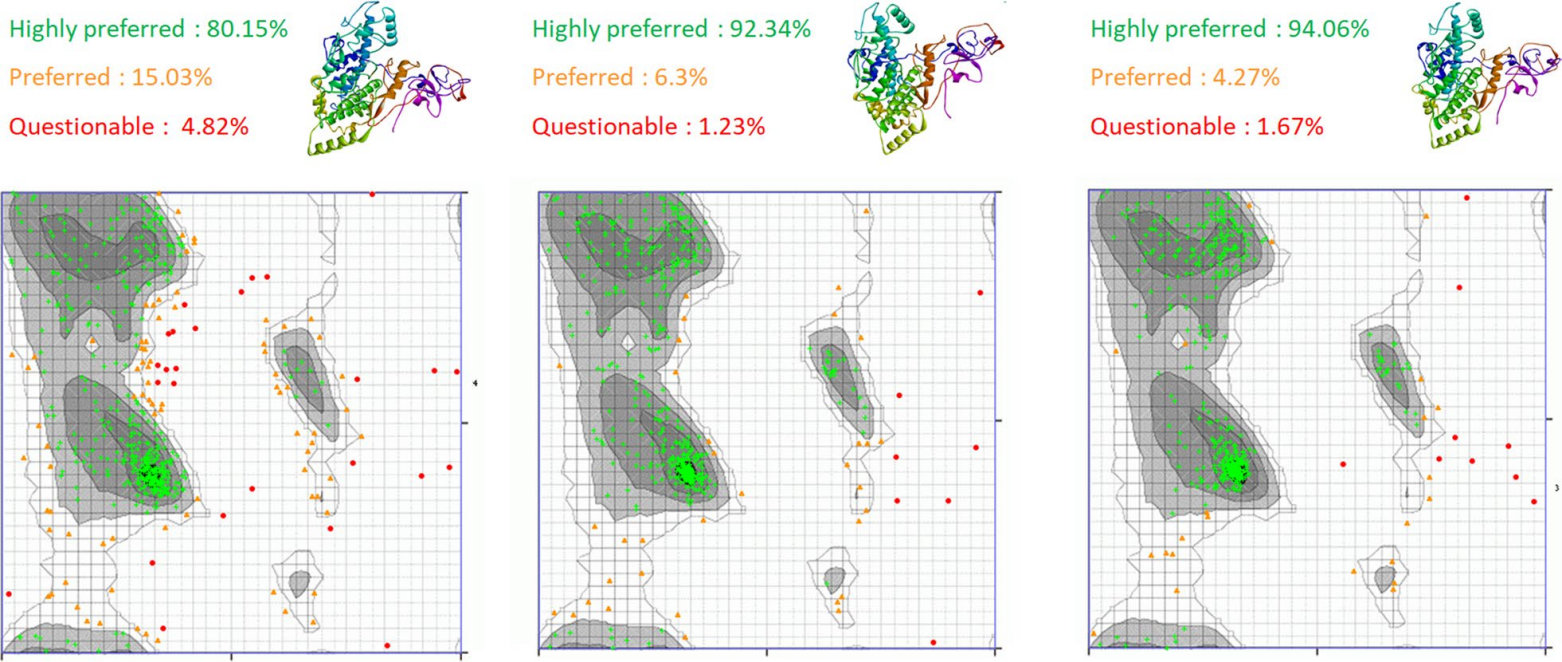
The modelled and refined structure of vaccine construct and validation via Ramachandran plot

The tertiary structure of the vaccine was chosen from the best 5 models; the structure chosen has a C-score of − 1.8 and an estimated RMSD value of 11.8 ± 4.5 Å. The model was validated through a Ramachandran plot. The amino acids are depicted as highly preferred observations, which are depicted in green with a cross symbol, and a total of 432 amino acids are located in the highly favourable regions, which account for 80.148% of the total. The preferred observations are shown in brown with a triangle symbol; a total of 81 amino acids are present in this favourable region, accounting for 15.028%, while 26 amino acids are shown in a red colour circle, accounting for 4.824%. To reduce these questionable observations, the tertiary structure is refined by the ModRefiner server and further meliorated by the GalaxyRefiner server. In this tertiary structure refined by ModRefiner, questionable observations are reduced to 7 (1.299%), highly favourable observations are increased to 498 (92.393%), and favourable observations are 34 (6.308%) indicating that after structure refinement most of the amino acids are falling in the highly favourable region and favourable as well as questionable observations are reduced by more than 2 times with compare to the raw tertiary structure modelled by I-TASSER. The GalaxyRefiner server was used to further improve the structure. The GalaxyRefiner server returns five refined models; the best model was selected. In this tertiary structure refined by the Galaxyrefiner, questionable observations are 9 (1.67%), highly favourable observations are increased to 507 (94.063%), and favourable observations are 23 (4.267%) (Fig. 3).

The comparison of the 3D model generated by AlphaFold 2 and I-TASSER adds value to the predicted structure. Although, the AlphaFold method is ranked top on the Critical Assessment of Structure Prediction (CASP) in 2018 and 2020, in this case it performed poorly. The rank-1 model generated has a pLDDT score of 48.80, less than 50, and a pTM score of 0.2790. I-TASSER's best model has a TM score of 0.50 ± 0.15, a C-score of − 1.81, and an RMSD value of 11.8 ± 4.5 Å. The multiple sequence alignment (MSA) coverage, the Local Distance Difference Test (LDDT) per position and alignment error are in Additional file 1.

### Docking and immune response analysis

**Fig. 4:**
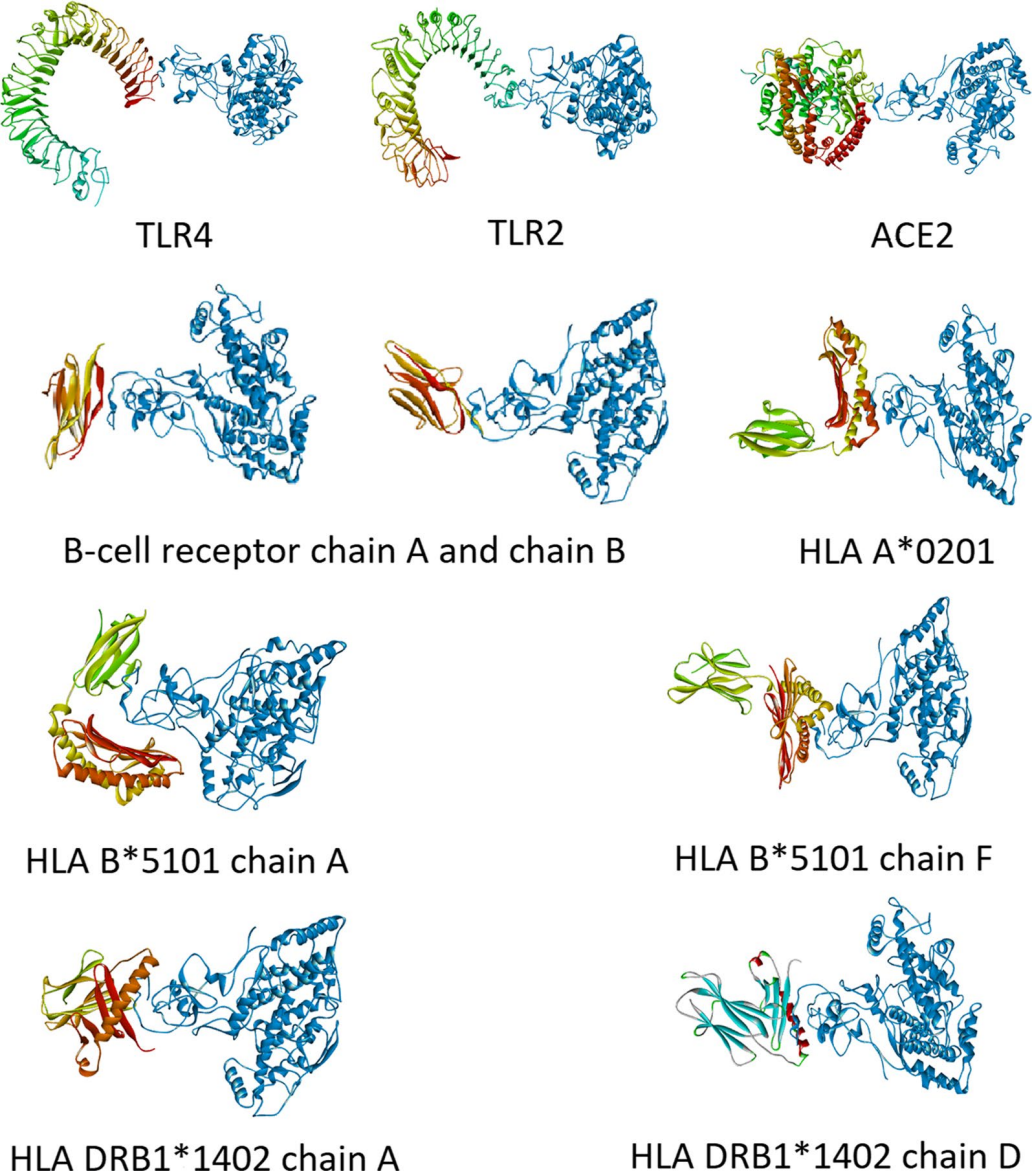
Interaction of vaccine construct (blue) with immune receptors and HLAs

Since the SARS-CoV-2 spike protein interacts with the ACE2 receptor, SARS-CoV-2 contact and transmission can be regulated by ACE2 receptor blockers. For docking of a vaccine with the ACE2 receptor, HADDOCK server clustered 173 structures into 6 clusters, which represent 86% of the water-refined models that the HADDOCK server generated. The best model has a z score of − 1.4 and a HADDOCK score of − 79.4 ± 2.6. For docking with TLR4, HADDOCK server clustered 199 structures into 3 clusters, which represent 99% of the water-refined models. Likewise, for TLR2, 168 structures in 7 clusters represent 84% of water refined models; for B-cell receptor chain A, 151 structures in 9 clusters represent 75% of water refined models; for B-cell receptor chain A, 164 structures in 8 clusters represent 82% of the water-refined models. Similarly, for HLA in which the lowest percentage of the water-refined model was 48% for HLA-B chain A and the highest was 81% for HLA-D chain A. Table 3 and Fig. 4 show the docking scores and docking interactions, respectively.

**Table 3:** Docking score of the receptor-vaccine construct complex

Docking score	ACE2	TLR4	TLR2	BCR chain A	BCR chain B	HLA A*0201	HLA B*5101 chain A	HLA B*5101 chain F	HLA DRB1*1402 chain A	HLA DRB1*1402 chain D
HADDOCK score	− 79.4 ± 2.6	− 133.4 ± 1.9	− 56.2 ± 1.7	− 82.1 ± 2.7	− 78.9 ± 3.7	− 66.8 ± 14.0	− 78.1 ± 12.4	− 43.7 ± 14.3	− 100.0 ± 8.8	− 108.2 ± 14.3
Cluster size	121	120	52	46	53	6	4	4	40	36
RMSD Lowest energy structure	0.90 ± 1.1	0.7 ± 0.5	0.9 ± 0.6	13.8 ± 0.3	0.8 ± 0.5	0.8 ± 0.5	0.6 ± 0.3	20.8 ± 0.1	1.0 ± 0.8	0.6 ± 0.3
Van der waals energy	− 70.0 ± 2.9	− 69.9 ± 6.6	− 44.6 ± 6.8	− 61.7 ± 3.0	− 49.1 ± 6.1	− 52.5 ± 3.0	− 84.0 ± 16.5	− 73.8 ± 4.5	− 84.5 ± 5.5	− 84.6 ± 5.9
Electrostatic energy	− 170.6 ± 13.5	− 214.4 ± 22.4	− 172.3 ± 21.2	− 24.4 ± 5.7	− 78.9 ± 12.8	− 172.8 ± 25.8	− 169.9 ± 26.6	− 94.2 ± 14.1	− 170.8 ± 33.7	− 181.2 ± 7.0
Desolvation energy	− 24.4 ± 2.4	− 53.0 ± 3.1	− 25.6 ± 3.9	− 48.0 ± 2.0	− 42.8 ± 2.8	− 21.3 ± 1.2	− 35.8 ± 4.2	− 41.8 ± 2.3	− 54.3 ± 2.4	− 50.5 ± 3.7
Restraints violation energy	490.4 ± 62.9	324.1 ± 55.2	483.9 ± 53.3	324.2 ± 44.7	287.2 ± 95.8	414.7 ± 82.1	756.5 ± 110.9	907.5 ± 158.8	729.9 ± 83.4	632.0 ± 80.2
Buried surface area	1878.9 ± 36.2	1802.9 ± 52.9	1558.1 ± 97.6	1530 ± 82.9	1457.3 ± 89.8	1933.2 ± 81.0	2895.4 ± 274.2	2251.8 ± 45.0	2457.0 ± 108.4	2427.0 ± 43.9
*Z*-score	− 1.4	− 1.4	− 1.7	− 1.4	− 1.7	− 1.0	− 1.9	− 1.7	− 1.5	− 1.5

**Fig. 5:**
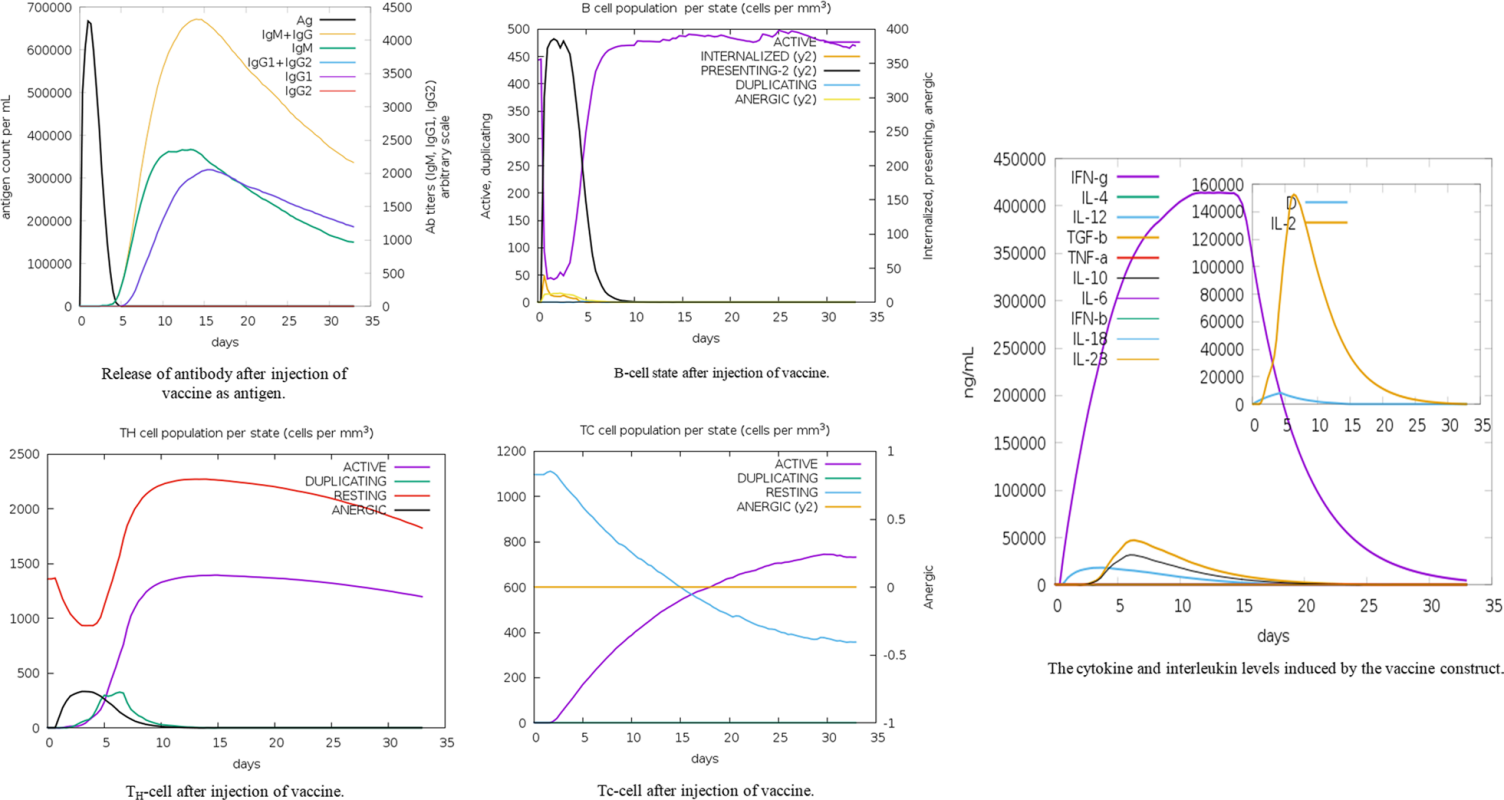
C-ImmSim simulation generated immune response after injection of vaccine construct as an antigen

After 5 days of administration, the immune response generated by the vaccine as an antigen shows a sharp increase in IgM and IgG antibodies, as well as IgG isotypes that help in neutralising the SARS-CoV-2 by binding with specific epitopes. In terms of cell activation, B cells exhibit high presenting activity for the first 5 days and a high level of activation within 0–10 days before reaching saturation, whereas TH and TC cells are activated exponentially within 5 days. It also stimulated memory cells. The cytokines released after administration show a high level of interferon-γ, with interleukin 2 coming in second. The disparities in both levels were significant. A comparison was made between the predictive vaccine and the currently available vaccine based upon the C-ImmSim simulation results by utilising the S-2P protein sequence shows the predictive vaccine response to be parallel with available vaccine. It showed better result in B cell response (Fig. 5).

## Discussion

In recent times, the focus of vaccine development is on sub-unit vaccines, as they are considered safer and more feasible to produce. Most vaccines for COVID-19 are mRNA vaccines, but due to the evolution of SARS-CoV-2, they are considering updating the sequence in the event that emerging variants may evade the protection provided by the existing vaccine. This is the limitation that can be solved by using sub-unit vaccines with multiple antigenic peptides. Epitope-based vaccines have a novel approach to generating a specific immune response to the desired antigen. Hence, epitopes were identified from the spike protein sequences of wild-type, delta, and omicron variants of SARS-CoV-2 to specifically target the spike protein of SARS-CoV-2, which is the primary factor responsible for the interaction with the host (human), as well as the emergences of new variants contributed by the mutations of the spike protein sequences. The previous vaccines for SARS-CoV were mostly based on RBD of spike protein, while in this study whole spike protein sequences were considered instead of domains. In this study, the number of B-cell epitopes obtained after screening was high, indicating favourable interactions between B-cells and the virus. Considering the moderate number of T-cell epitopes obtained and the immune response generated, cell-mediated immunity was high and long lasting. The comparison made between predictive vaccine and S-2P mutant favours the interaction of B-cells with virus. Another important property of the vaccine was that it was less homologous to human proteins (17%), thus the chance of molecular mimicry and cross-reactivity was very low, providing the vaccine construct with no autoimmunity. The vaccine constructed was docked with immune receptors. The results indicate that the vaccine was interacting with TLR4 and TLR2, which induce an innate immune response. The vaccine was also interacting with B-cell receptors, which can elicit cellular or hormonal immune responses, while the interaction of the vaccine with the ACE2 receptor can lead to the blocking of the ACE2 receptor, which is responsible for the interaction and entry of the virus into the host (human). Further docking and experimental validation are required to confirm the above interactions and the overall effect of these interactions within the host.

## Conclusion

In the present study, the vaccine construct was based on spike proteins of wild-type Delta and Omicron. Several immuno-informatics tools are used to propose potentially antigenic and immunogenic epitopes, which will further help to design vaccines of the next generation. Omicron has the highest mutation in spike proteins, and the delta variant was, however, responsible for the second wave and high rate of effectiveness of COVID-19. The vaccine construct is based upon major SARS-Cov-2 variants to combat emerging variants like the XE virus, which is a combination of delta and omicron strains. The vaccine is constructed using 18-B-cell epitopes and 11-T-cell epitopes. Out of the total construct, ‘MSLGVENSV’ was found to be a novel epitope that may be used to generate neutralising antibodies for detection and neutralisation purposes. Although synthetically modified peptides have excellent prospects, as they have potential to improving stability and bioavailability. Experimental authentication is further required for validation of the outcome of the study. However, the vaccine was stable and found to interact with immune receptors and HLAs as well as with ACE-2 receptors, clearly indicating the potential for inducing humoral and cell-mediated immune responses. The C-ImmSim simulation observation strengthens the information like antibody release, cytokines release, and the state of B and T cells upon a single injection of vaccine as an antigen sufficient to provide immunity. Peptide-based therapeutics have various benefits over conventional molecules. Although repurposed medications as well as vaccines will serve as a topline of safeguard against COVID-19, future side effects of vaccination as well as resistance development as a consequence of drug administration also provide space for advancements in therapeutics.

## Supplementary Information

**Additional file 1.** Additional information supporting the study.

## Data Availability

Not applicable.

## Abbreviations

ACE2: Angiotensin Converting Enzyme 2
ANN: Artificial Neural Network
BLAST: Basic Local Alignment Search Tool
CASP: Critical Assessment of Structure Prediction
COVID-19: Coronavirus Disease
HADDOCK: High Ambiguity Driven protein–protein DOCKing
IFN: Interferons
I-TASSER: Iterative Threading ASSEmbly Refinement
IEDB: Immune Epitope DataBase
LDDT: Local Distance Difference Test
MSA: Multiple sequence alignment
NTD: N-terminal domain
PDB: Protein data bank
RBD: Receptor Binding Domain
SARS-CoV-2: Severe Acute Respiratory Syndrome Coronavirus-2
SARS-CoV: Severe Acute Respiratory Syndrome related Coronavirus
SOPMA: Self-optimised prediction method with alignment
SVM: Support vector mechanism
TLR2: Toll like receptor 2
TLR4: Toll like receptor 4
VOC: Variant of concern
WHO: World Health Organisation

## References

[1] Almofti YA, Abd-elrahman KA, Eltilib EEM (2021). Vaccinomic approach for novel multi epitopes vaccine against severe acute respiratory syndrome coronavirus-2 (SARS-CoV-2). BMC Immunol.

[2] Harvey WT (2021). SARS-CoV-2 variants, spike mutations and immune escape. Nat Rev Microbiol.

[3] Bayarri-Olmos R (2021). Functional effects of receptor-binding domain mutations of SARS-CoV-2 B.1.351 and P.1 variants. Front Immunol.

[4] Sanches PRS (2021). Recent advances in SARS-CoV-2 Spike protein and RBD mutations comparison between new variants Alpha (B.1.1.7, United Kingdom), Beta (B.1.351, South Africa), Gamma (P.1, Brazil) and Delta (B.1.617.2, India). J Virus Erad.

[5] Shiehzadegan S, Alaghemand N, Fox M, Venketaraman V (2021). Analysis of the delta variant B.1.617.2 COVID-19. Clin Pract.

[6] Motozono C (2021). SARS-CoV-2 spike L452R variant evades cellular immunity and increases infectivity. Cell Host Microbe.

[7] Starr TN, Greaney AJ, Dingens AS, Bloom JD (2021). Complete map of SARS-CoV-2 RBD mutations that escape the monoclonal antibody LY-CoV555 and its cocktail with LY-CoV016. Cell Rep Med..

[8] Mccallum M et al. (2021) Variant of concern, vol 654, no August, pp 648–654

[9] Andrews N (2022). Covid-19 vaccine effectiveness against the Omicron (B.1.1.529) variant. N Engl J Med.

[10] Saha S, Raghava GPS (2006). AlgPred: prediction of allergenic proteins and mapping of IgE epitopes. Nucleic Acids Res..

[11] Paul S, Sidney J, Sette A, Peters B (2016). TepiTool: a pipeline for computational prediction of T cell epitope candidates. Curr Protoc Immunol.

[12] Paul S (2015). Development and validation of a broad scheme for prediction of HLA class II restricted T cell epitopes. J Immunol Methods.

[13] Doytchinova IA, Flower DR (2007). VaxiJen: a server for prediction of protective antigens, tumour antigens and subunit vaccines. BMC Bioinform.

[14] Gupta S, Kapoor P, Chaudhary K, Gautam A, Kumar R, Raghava GPS (2013). In silico approach for predicting toxicity of peptides and proteins. PLoS ONE.

[15] Calis JJA (2013). Properties of MHC class I presented peptides that enhance immunogenicity. PLoS Comput Biol.

[16] Dhanda SK (2018). Predicting HLA CD4 immunogenicity in human populations. Front Immunol..

[17] Dhanda SK, Vir P, Raghava GPS (2013). Designing of interferon-gamma inducing MHC class-II binders. Biol Direct.

[18] Bui HH, Sidney J, Li W, Fusseder N, Sette A (2007). Development of an epitope conservancy analysis tool to facilitate the design of epitope-based diagnostics and vaccines. BMC Bioinform.

[19] Bui HH, Sidney J, Dinh K, Southwood S, Newman MJ, Sette A (2006). Predicting population coverage of T-cell epitope-based diagnostics and vaccines. BMC Bioinform.

[20] Kim J, Yang YL, Jang SH, Jang YS (2018). Human β-defensin 2 plays a regulatory role in innate antiviral immunity and is capable of potentiating the induction of antigen-specific immunity. Virol J.

[21] Kim J, Yang YL, Jang YS (2019). Human β-defensin 2 is involved in CCR2-mediated Nod2 signal transduction, leading to activation of the innate immune response in macrophages. Immunobiology.

[22] Dhanda SK (2018). Development of a novel clustering tool for linear peptide sequences. Immunology.

[23] Buchan DWA, Jones DT (2019). The PSIPRED protein analysis workbench: 20 years on. Nucleic Acids Res.

[24] Jones DT (1999). Protein secondary structure prediction based on position-specific scoring matrices. J Mol Biol.

[25] Xu D, Zhang Y (2011). Improving the physical realism and structural accuracy of protein models by a two-step atomic-level energy minimization. Biophys J.

[26] Yang J, Yan R, Roy A, Xu D, Poisson J, Zhang Y (2014). The I-TASSER suite: protein structure and function prediction. Nat Methods.

[27] Yang J, Zhang Y (2015). I-TASSER server: new development for protein structure and function predictions. Nucleic Acids Res.

[28] Zheng W, Zhang C, Li Y, Pearce R, Bell EW, Zhang Y (2021). Folding non-homologous proteins by coupling deep-learning contact maps with I-TASSER assembly simulations. Cell Rep Methods.

[29] Anderson RJ, Weng Z, Campbell RK, Jiang X (2005). Main-chain conformational tendencies of amino acids. Proteins Struct Funct Genet.

[30] Ko J, Park H, Heo L, Seok C (2012). GalaxyWEB server for protein structure prediction and refinement. Nucleic Acids Res.

[31] Van Zundert GCP (2016). The HADDOCK2.2 Web Server: user-friendly integrative modeling of biomolecular complexes. J Mol Biol.

[32] Honorato RV (2021). Structural biology in the clouds: the WeNMR-EOSC ecosystem. Front Mol Biosci.

[33] Qin S, Zhou HX (2007). Meta-PPISP: a meta web server for protein-protein interaction site prediction. Bioinformatics.

[34] Castiglione F, Deb D, Srivastava AP, Liò P, Liso A (2021). From infection to immunity: understanding the response to SARS-CoV2 through in-silico modeling. Front Immunol.

[35] Rapin N, Lund O, Bernaschi M, Castiglione F (2010). Computational immunology meets bioinformatics:the use of prediction tools for molecular binding in the simulation of the immune system. PLoS One.

