# Supplementary material for: Cross-variant proof predictive vaccine design based on SARS-CoV-2 spike protein using immunoinformatics approach
Source: Beni Suef Univ J Basic Appl Sci. 2023 Jan 10;12(1):5. doi: 10.1186/s43088-023-00341-4 (PMC9831375; doi:10.1186/s43088-023-00341-4)
Supplement: Supplementary file 1 — Additional file 1. Additional information supporting the study. [file 43088_2023_341_MOESM1_ESM.docx]

**The B-cell and T-cell epitopes**

Table 1 The B-cell epitopes with conservancy analysis of wild type, delta and omicron.

| S.No. | Sequence | Length | Antigenecity | Percent of protein sequence matches at identity <= 100% | Minimum identity | Maximum identity |
| --- | --- | --- | --- | --- | --- | --- |
| 1. | NKSWMESEFR | 10 | 0.6813 | 100.00% (3/3) | 100.00% | 100.00% |
| 2. | LREFVFKNID | 10 | 0.8332 | 100.00% (3/3) | 100.00% | 100.00% |
| 3. | PDKVFRSSVLHS | 12 | 1.649 | 100.00% (3/3) | 100.00% | 100.00% |
| 4. | GINITRFQTLLALHRSYLTP | 20 | 0.6602 | 100.00% (3/3) | 100.00% | 100.00% |
| 5. | KTQSLLIVNN | 10 | 0.8332 | 100.00% (3/ 3) | 100.00% | 100.00% |
| 6. | MDLEGKQGNFKN | 12 | 1.3296 | 100.00% (3/3) | 100.00% | 100.00% |
| 7. | KNIDGYFKIYSKHTPINL | 18 | 0.749 | 66.67% (2/3) | 88.89% | 100.00% |
| 8. | FLGVYYHKNNKSWMESEFRV | 20 | 0.5741 | 33.33% (1/3) | 70.00% | 100.00% |
| 9. | LGDIAARDLI | 10 | 0.9922 | 100.00% (3/3) | 100.00% | 100.00% |
| 10. | HADQLTPTWR | 10 | 0.6323 | 100.00% (3/3) | 100.00% | 100.00% |
| 11. | TSALLAGTIT | 10 | 0.6493 | 100.00% (3/3) | 100.00% | 100.00% |
| 12. | IAYTMSLGAE | 10 | 1.0165 | 66.67% (2/3) | 90.00% | 100.00% |
| 13. | SIAIPTNFTISVTT | 14 | 0.8408 | 100.00% (3/3) | 100.00% | 100.00% |
| 14. | EIRASANLAATKMS | 14 | 0.8607 | 100.00% (3/3) | 100.00% | 100.00% |
| 15. | KRVDFCGKGYHLMS | 14 | 1.0454 | 100.00% (3/3) | 100.00% | 100.00% |
| 16. | MSLGAENSVAYSNN | 14 | 0.8278 | 66.67% (2/3) | 92.86% | 100.00% |
| 17. | IPFAMQMAYRFNGIGVTQ | 18 | 1.4137 | 100.00% (3/3) | 100.00% | 100.00% |
| 18. | PTNFTISVTTEILPVSMTKT | 20 | 1.2666 | 100.00% (3/3) | 100.00% | 100.00% |

Table 2 MHC class I epitopes.

| **Peptide** | **IC50 value** | **Immunogenicity score** | **Antigenicity** | **Alleles**  **(HLA)** |
| --- | --- | --- | --- | --- |
| KWPWYIWLG | 186.87 | 0.52143 | 1.0478 | A24 |
| NRALTGIAV | 46.43 | 0.20642 | 0.5302 | B39 |
| IAIVMVTIM | 21.79 | 0.06312 | 1.1339 | B44 |
| AEIRASANL | 9.62 | 0.00689 | 0.7082 | B58 |
| MSLGVENSV | 466.42 | 0.8819 | 0.06283 | B58 |

Table 3 MHC class II epitopes.

| **Peptide** | **Median consensus percentile** | **Antigenicity** | **IFN-gamma** | **IFN-gamma score** |
| --- | --- | --- | --- | --- |
| IPFAMQMAYRFNGIG | 6.5 | 1.2828 | Negative | -0.40418676 |
| SNLKPFERDISTEIY | 15 | 0.8255 | Negative | -0.039052853 |
| AEIRASANLAATKMS | 13 | 0.8255 | Negative | -0.29898458 |
| IGINITRFQTLLALH | 18 | 0.8391 | Negative | -0.44508838 |
| WYIWLGFIAGLIAIV | 18 | 0.577 | Positive | 0.92824136 |
| IWLGFIAGLIAIVMV | 18 | 0.615 | Positive | 0.76829436 |

**Population coverage analysis**

Table 4 The population coverage analysis for MHC Class I and MHC class II for the world population.

| Population/area | Class I | | | Class II | | | Class combined | | |
| --- | --- | --- | --- | --- | --- | --- | --- | --- | --- |
|  | Coverage^a^ | Average hit^b^ | Pc90^c^ | Coverage^a^ | Average hit^b^ | Pc90^c^ | Coverage^a^ | Average hit^b^ | Pc90^c^ |
| World | 88.42% | 16.61 | 9.5 | 49.02% | 5.99 | 2.16 | 94.1% | 22.6 | 12.78 |
| Average | 88.42 | 16.61 | 9.5 | 49.02 | 5.99 | 2.16 | 94.1 | 22.6 | 12.78 |
| Standard deviation | 0.0 | 0.0 | 0.0 | 0.0 | 0.0 | 0.0 | 0.0 | 0.0 | 0.0 |

^a^ Projected population coverage

^b^ Average number of epitope hits / HLA combinations recognised by population

^c^ Minimum number of epitope hits / HLA combination recognised by 90% of the population

**Predictive vaccine**

FASTA sequence of predictive vaccine and a separate pdb file for 3D model.

>PREDICTIVE_VACCINE

MRVLYLLFSFLFIFLMPLPGVFGGIGDPVTCLKSGAICHPVFCPRRYKQIGTCGLPGTKC

CKKPEAAAKWYIWLGFIAGLIAIVKKIPFAMQMAYRFNGIGKKSNLKPFERDISTEIYKK

AEIRASANLAATKMSKKIGINITRFQTLLALHKKNKSWMESEFRKKPTNFTISVTTEILP

VSMTKTKKEIRASANLAATKMSKKLREFVFKNIDKKPDKVFRSSVLHSKKGINITRFQTL

LALHRSYLTPKKKTQSLLIVNNKKLGDIAARDLIKKHADQLTPTWRKKTSALLAGTITKK

IAYTMSLGAEKKKNIDGYFKIYSKHTPINLKKMDLEGKQGNFKNKKKRVDFCGKGYHLMS

KKMSLGAENSVAYSNNKKIPFAMQMAYRFNGIGVTQGPGPGKWPWYIWLGGPGPGNRALT

GIAVGPGPGIAIVMVTIMGPGPGAEIRASANLGPGPGMSLGVENSVEAAAKMRVLYLLFS

FLFIFLMPLPGVFGGIGDPVTCLKSGAICHPVFCPRRYKQIGTCGLPGTKCCKKPHHHHH

H

**Secondary structure prediction
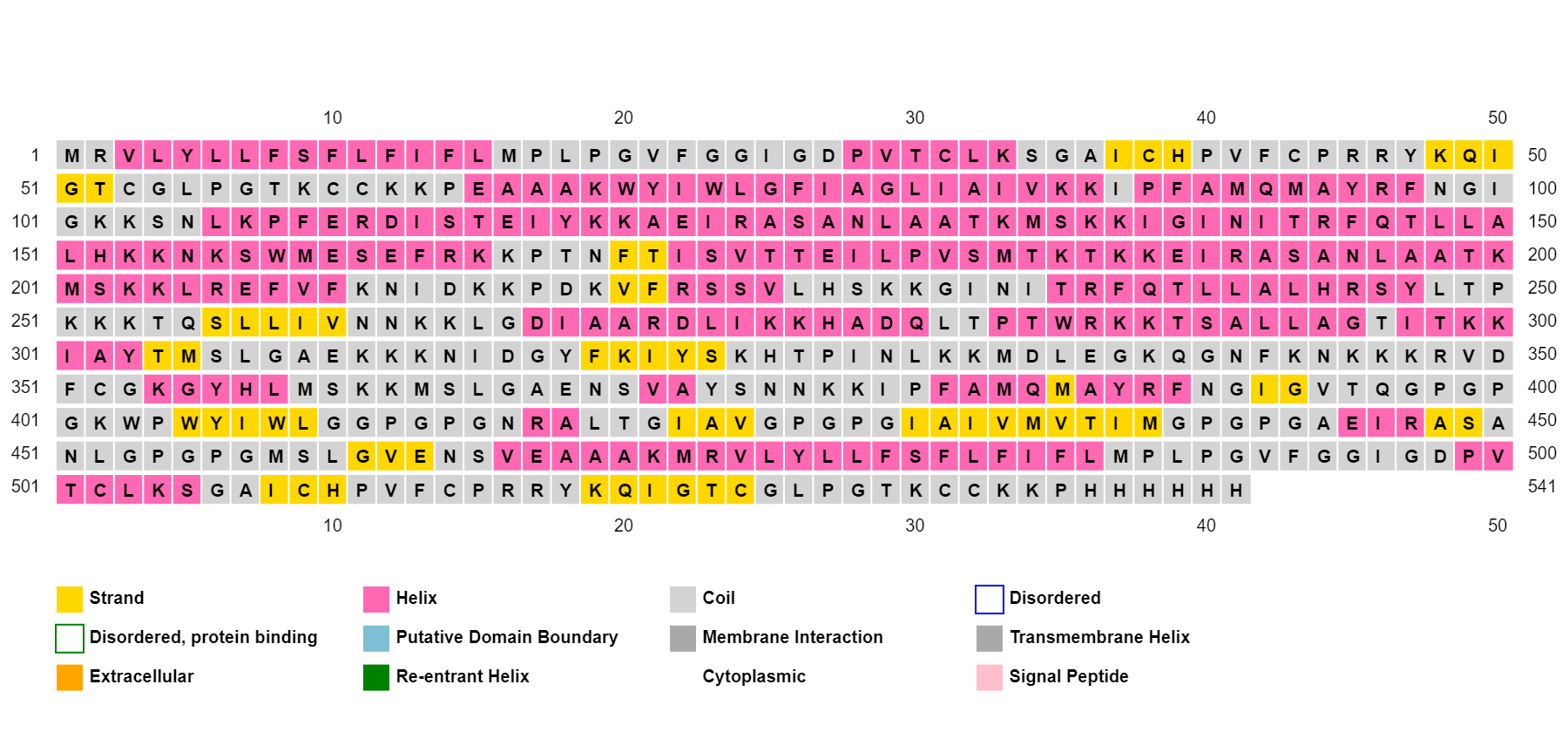
**

Fig. 1Amino acids forming the secondary structure obtained via PSIPRED.

**
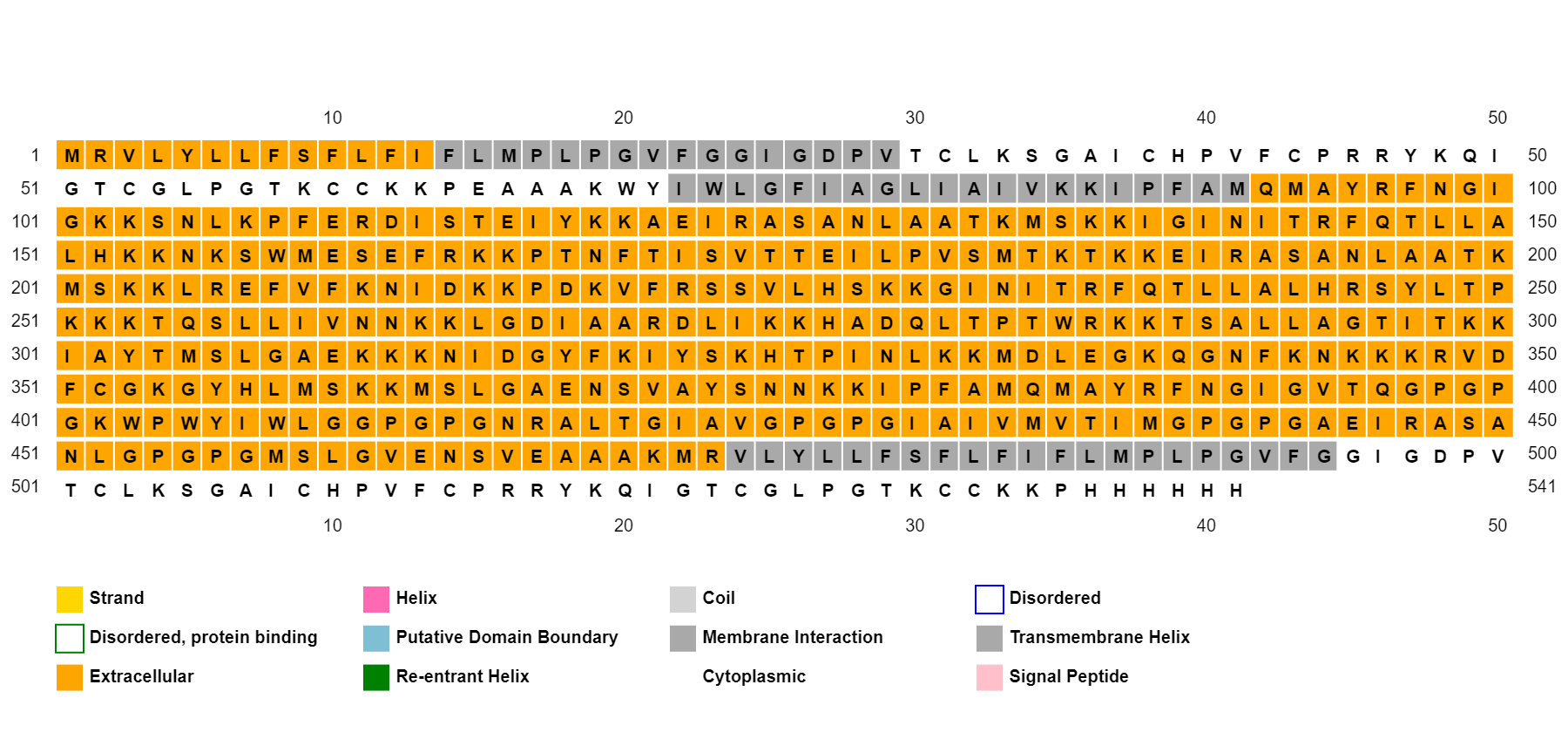
**

Fig. 2 Nature of amino acids indicating to the probable cellular location of protein obtained via PSIPRED.

**
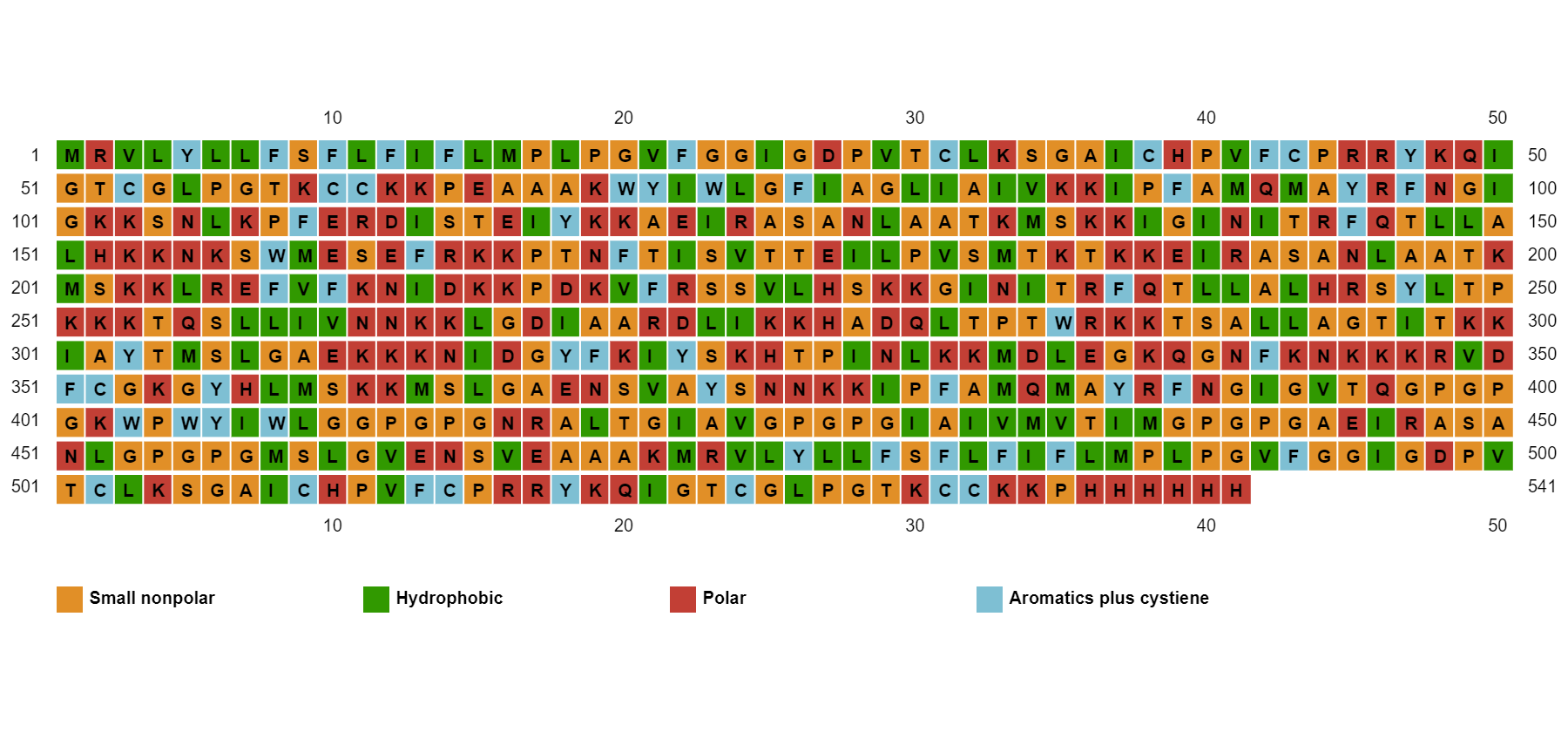
**

Fig. 3 Basic nature of amino acid forming the vaccine obtained via PSIPRED.

**Alphafold model**


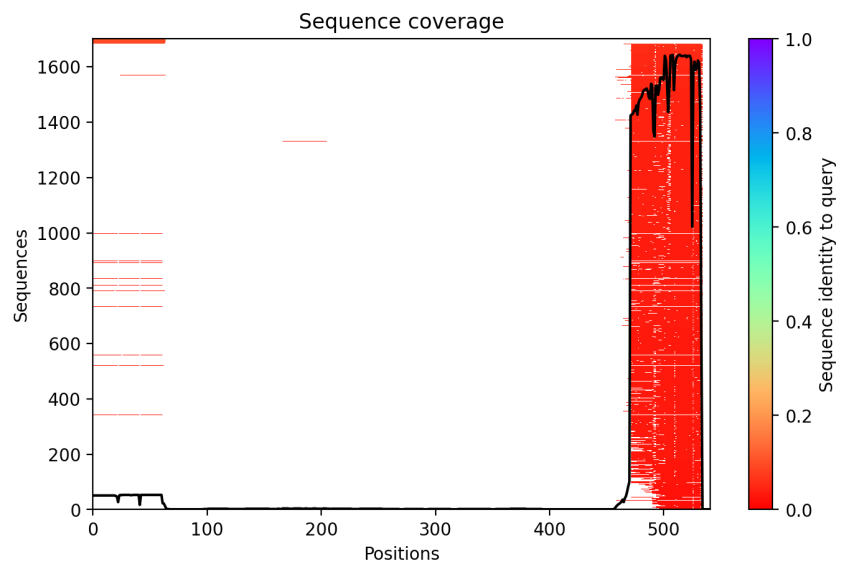


Fig. 4 Sequence coverage of predictive vaccine sequence by Alphafold for multiple sequence alignment.


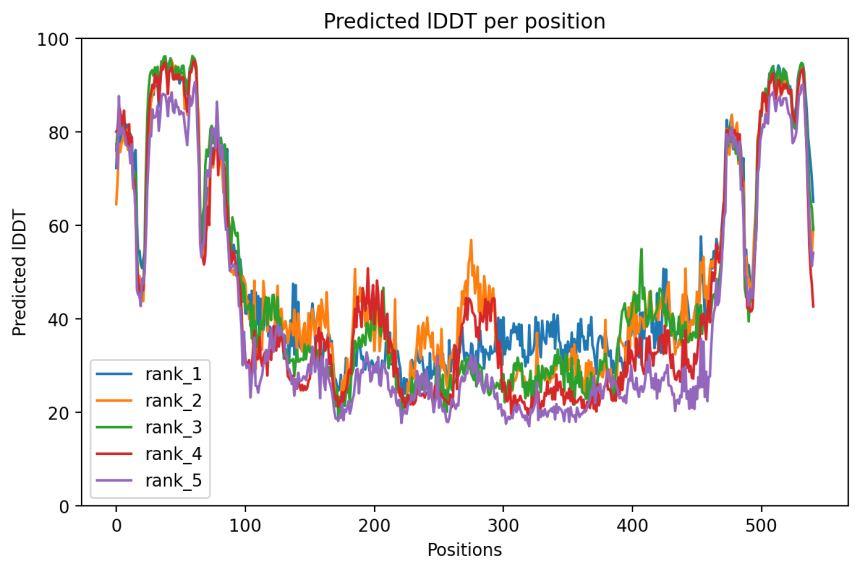


Fig. 5 Predicted LDDT per position.


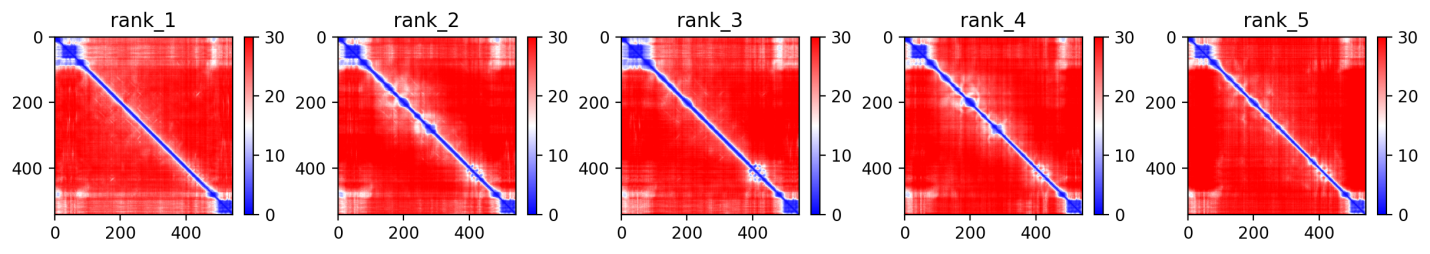


Fig. 6 Predicted alignment error plot for models.

**Immune response analysis**

b)

a)

c)

Fig. 7 The memory cell population of a) lymphocyte B cells, b) lymphocyte helper (T_H_ ) cells, and c) lymphocyte T cytotoxic (T_c_ ) cells.

b)

a)

d)

c)

Fig. 8 The cell population and their state after administration of the vaccine as an antigen a) natural killer (NK) cells, b) macrophage (MA), c) dendritic (DC) cells, and d) epithelial (EP) cells.

**C-ImmSim simulation comparison: S-2P mutant and predictive vaccine**

The Pfizer and Moderna encoded surface glycoprotein (S-2P mutant) of SARS-CoV-2 through the RNA vaccine. This S-2P mutant surface glycoprotein is the main antigen that generates immunity in humans. So, here in the C-ImmSim simulation, a comparison between S-2P and a predictive vaccine was done. To compare, the S-2P mutant was given as an antigen to generate an immune response. The same was done for the predictive vaccine.

The antibody tiers released by S-2P and predictive vaccine are similar, except that IgM was released more in the case of S-2P whereas IgG1 was released more in the case of predictive vaccine. There is no difference in the release of cytokines and interleukins. The T-cell response was relatively better in the case of S-2P, while the B-cell response was relatively better in the case of the predictive vaccine. In conclusion, the predictive vaccine is comparable to the available vaccine.


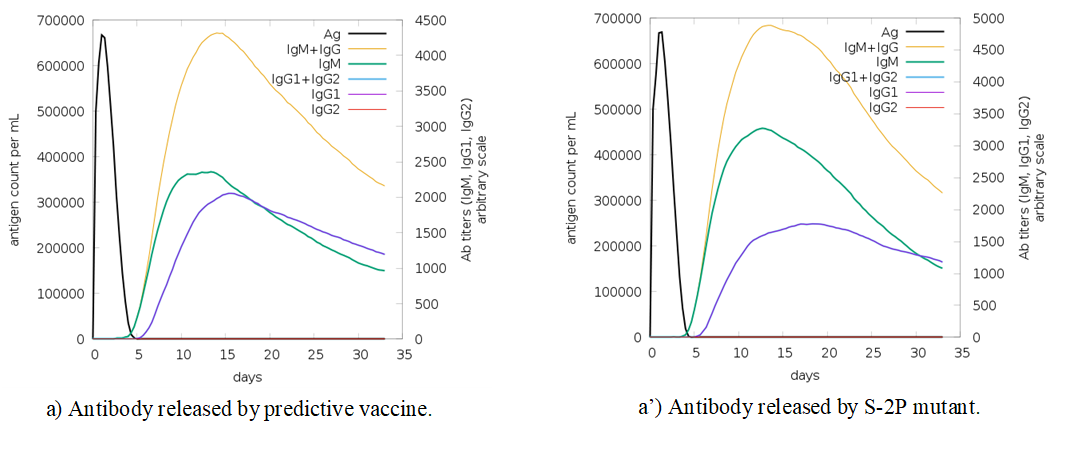


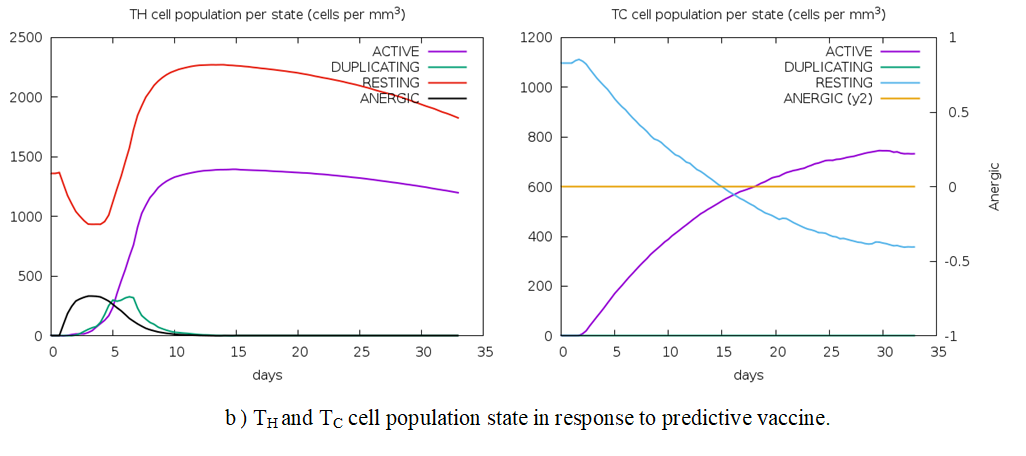


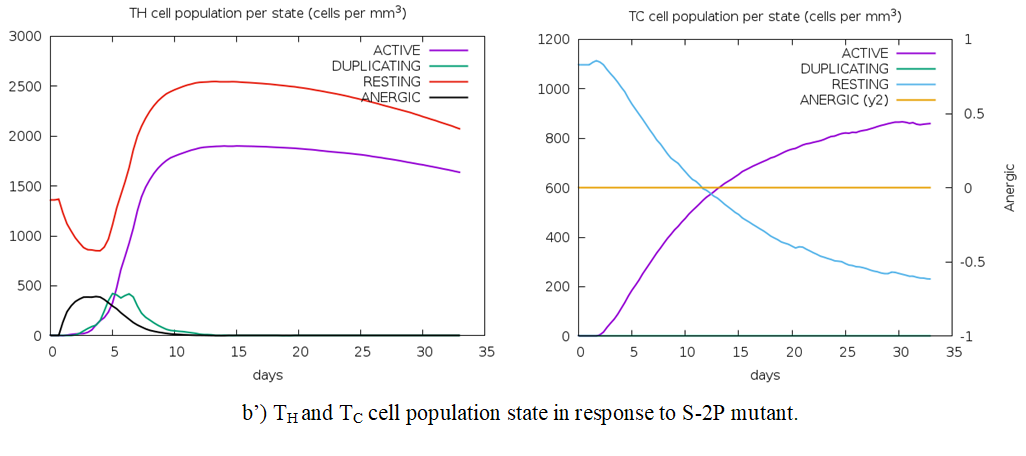


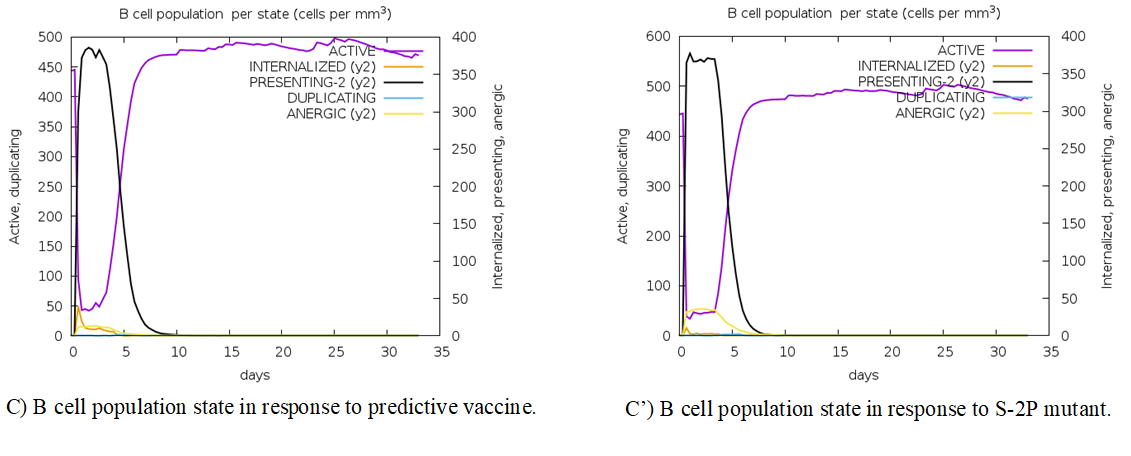


Fig. 9 C-ImmSim simulation results of a) antibody release, b) T cell state, and c) B cell state for comparison of the S-2P mutant and the predictive vaccine response when given as an antigen.
